# Three-dimensional evaluation of the coccyx movement between supine and standing positions using conventional and upright computed tomography imaging

**DOI:** 10.1038/s41598-021-86312-0

**Published:** 2021-03-25

**Authors:** Fumiko Yagi, Yoshitake Yamada, Minoru Yamada, Yoichi Yokoyama, Kiyoko Mukai, Takehiro Nakahara, Keiichi Narita, Masahiro Jinzaki

**Affiliations:** 1 Department of Radiology, Keio University School of Medicine, 35 Shinanomachi, Shinjuku-ku, Tokyo 160-8582 Japan; 2 Department of Radiology, St. Luke’s International Hospital, 9-1 Akashi-cho, Chuo-ku, Tokyo Japan

**Keywords:** Anatomy, Musculoskeletal system, Skeleton

## Abstract

Currently, no three-dimensional reference data exist for the normal coccyx in the standing position on computed tomography (CT); however, this information could have utility for evaluating patients with coccydynia and pelvic floor dysfunction. Thus, we aimed to compare coccygeal parameters in the standing versus supine positions using upright and supine CT and evaluate the effects of sex, age, and body mass index (BMI) on coccygeal movement. Thirty-two healthy volunteers underwent both upright (standing position) and conventional (supine position) CT examinations. In the standing position, the coccyx became significantly longer and straighter, with the tip of the coccyx moving backward and downward (all *p* < 0.001). Additionally, the coccygeal straight length (standing/supine, 37.8 ± 7.1/35.7 ± 7.0 mm) and sacrococcygeal straight length (standing/supine, 131.7 ± 11.2/125.0 ± 10.7 mm) were significantly longer in the standing position. The sacrococcygeal angle (standing/supine, 115.0 ± 10.6/105.0 ± 12.5°) was significantly larger, while the lumbosacral angle (standing/supine, 21.1 ± 5.9/25.0 ± 4.9°) was significantly smaller. The migration length of the tip of the coccyx (mean, 7.9 mm) exhibited a moderate correlation with BMI (*r* = 0.42, *p* = 0.0163). Our results may provide important clues regarding the pathogenesis of coccydynia and pelvic floor dysfunction.

## Introduction

The coccyx comprises the terminal vertebral segments of the human vertebral column and provides weight-bearing support in the sitting position and positional support to the anus. Many ligaments and muscles are attached to the coccyx, supporting the pelvic floor and contributing to voluntary bowel control^1^. Previous studies have shown that assessments of coccygeal movement play an important part in evaluations of coccydynia^2^ and pelvic floor dysfunction^3^. Some authors^4,5^ have used plain radiography to demonstrate that individuals with greater ventral angulation of the coccyx are at a higher risk of developing idiopathic coccydynia. In addition, coccydynia has been reported to be aggravated by standing and/or walking^6^. Furthermore, it has been reported that the development of coccydynia is related to both obesity and vaginal delivery due to luxation and hypermobility^7,8^. However, in previous studies, the coccyx was assessed only by plain radiography in the standing and sitting positions^2,7,8^, computed tomography (CT)/magnetic resonance imaging (MRI) in the supine position^9–16^, or MRI in the sitting position^17^.

Since humans spend the majority of their time in the upright position, and the effects of gravity and intrapelvic pressure are reduced in the supine position, understanding the morphology, morphometry, and movement of the coccyx in the standing position likely has clinical utility. However, to the best of our knowledge, no reference data for the normal coccyx in the standing position on CT imaging exist at present. In addition, as far as we know, no clinical studies to date have compared coccygeal morphology in the supine versus standing positions. Recently, upright CT has been developed based on conventional 320-detector row CT to clarify the effects of gravity on the human body^18^.

Thus, this study aimed to compare coccygeal parameters in the standing versus supine positions using upright and supine CT imaging and to evaluate the effects of sex, age, and body mass index (BMI) on coccygeal movement in healthy volunteers.

## Methods

This prospective study was approved by the Keio University School of Medicine Ethics Committee (#20160384), and written informed consent was obtained from all participants. All methods were performed in accordance with the relevant guidelines and regulations. Asymptomatic male and female volunteers were recruited from a volunteer recruitment company. To evaluate normal whole-body anatomy, volunteers were excluded if they had a history of smoking, diabetes, hypertension, dyslipidemia, an awareness of dysuria, vertebral pain, diseases of the vertebral column, or its developmental defects, as well as those who had undergone surgeries or were currently undergoing treatment. A total of 32 healthy volunteers (16 men and 16 women; with four men and four women in the third, fourth, fifth, and sixth decades of life, mean age 48.4 ± 11.5 years [range, 30–68 years]) participated in this study conducted between April 2018 and October 2018. Among the 16 women, nine had given birth (all vaginal deliveries) and seven had not. The 32 enrolled volunteers had been analyzed for different purposes in previous studies that evaluated the vena cava, aorta, and pelvic floor^19^ as well as the brain^20^ and lung volume^21,22^ but did not evaluate movement of the coccyx. All volunteers prospectively underwent conventional 320-detector row CT imaging (supine CT) (Aquilion ONE, Canon Medical Systems Corporation, Japan) in the supine position, as well as upright CT imaging (prototype TSX-401R, Canon Medical Systems Corporation, Japan)^18–22^ in the standing position on the same day.

To maintain urinary bladder tension, volunteers were instructed to refrain from urinating before undergoing CT. Body trunk scans were acquired in the standing and supine positions. Scanning was performed at 120 kVp, with 0.5 s of gantry rotation, a helical scan mode (80-row detector), a noise index of 15 for 5 mm, and a helical pitch of 0.8 for abdominal CT. Image reconstruction was performed using Adaptive Iterative Dose Reduction 3D^23^.

**Figure 1:**
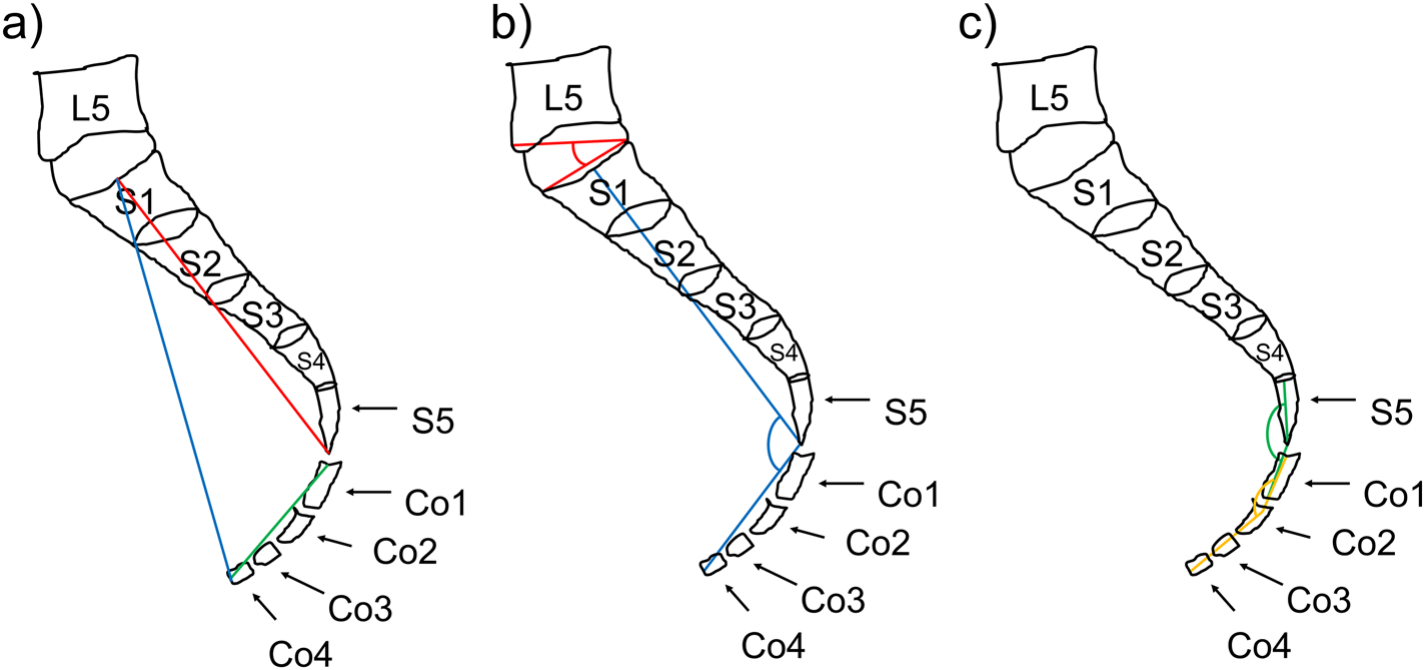
Coccygeal parameters. (**a**) Straight lengths. Green, coccygeal straight length; red, sacral straight length; blue, sacrococcygeal straight length. (**b**) Angles of the sacrum. Red, lumbosacral angle; blue, sacrococcygeal angle. (**c**) Angles of the coccyx. Green, sacrococcygeal joint angle; orange, intercoccygeal angle.

**Figure 2:**
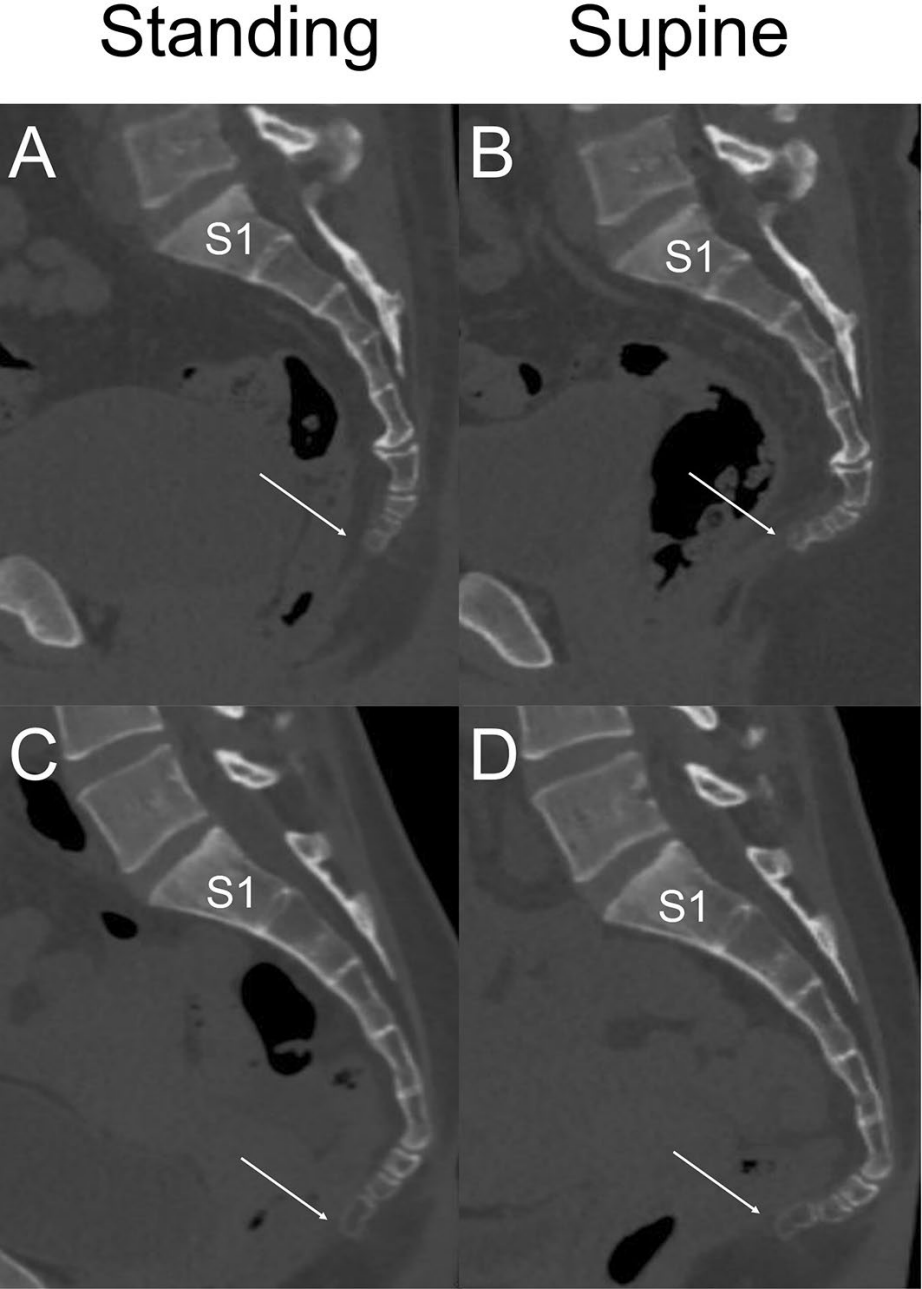
Computed tomography imaging of the coccyx in healthy volunteers. The coccyx of a 36-year-old man in the standing position (**A**) and the supine position (**B**), and the coccyx of a 47-year-old parous woman in the standing position (**C**) and supine position (**D**) in the mid-sagittal plane. As demonstrated, the coccyx becomes longer and straighter, with the tip of the coccyx (white arrow) moving backward and downward relative to the supine position.

CT images were processed and analyzed by the SYNAPSE VINCENT image analysis system (Fujifilm Inc.), and qualitative and quantitative parameters of the coccyx were assessed. Table 1 and Figs. 1 and 2 describe the coccygeal parameters documented in this study^11,12^. The two types of CT images underwent rigid registration with reference to the sacral vertebra, S1. The migration length of the tip of the coccyx in the standing to supine position was calculated by ((x2  − x1)^2 + (y2 − y1)^2 + (z2 − z1)^2)^(1/2), where the coordinates of the tip of the coccyx in the standing position were x1, y1, and z1, and the coordinates of the tip of the coccyx in the supine position were x2, y2, and z2. Measurements were repeated in the first 16 of the 32 subjects after an interval of one month to assess intra-observer repeatability; measurements were also repeated by another independent observer to determine inter-observer reproducibility.

**Table 1:** Definitions/descriptions of parameters of the morphology and morphometry of the coccyx.

Parameter	Definition/description
Coccygeal straight length^11,12^	Measured in a straight line from the middle of the upper border of Co1 to the coccygeal tip (Fig. 1a)
Sacral straight length^11,12^	Measured in a straight line from the middle of the upper border of S1 to the middle of the inferior border of S5 (Fig. 1a)
Sacrococcygeal straight length^11,12^	Measured from S1 to the tip of the coccyx using the same methods as applied to the coccyx (Fig. 1a)
Lumbosacral angle^11^	Measured using parallel lines, one to the superior surface of the first sacral segment and a second to the horizontal plane (Fig. 1b)
Sacrococcygeal angle^11^	Formed by the intersection of a line between the midpoint of the upper borders of S1 and Co1 and a line between the latter and the tip of the coccyx (Fig. 1b)
Sacrococcygeal joint angle^11^	Formed between lines intersecting the middle of S5 and Co1 (Fig. 1c)
Intercoccygeal angle^11^	Formed between lines intersecting the middle of the first and last coccygeal segments in the median plane (Fig. 1c)
Migration length	Migration length of the tip of the coccyx between the supine and standing positions when both images were matched for S1 as a standard point

Data are presented as mean ± standard deviation (SD). Data were tested for normality using the Shapiro-Wilk test. Mean differences between the standing and supine positions were compared using paired *t-*tests. Comparisons between men and women, as well as between women who had or had not experienced childbirth, were performed using the Mann-Whitney U test. Correlations between migration length and age, BMI, height, and body weight were assessed using Pearson’s correlation analyses. The strength of the correlations was defined as follows according to the Cohen classification^24^: weak, 0.10 ≤|*r*|< 0.30; moderate, 0.30 ≤|*r*|< 0.50; and strong, 0.50 ≤|*r*|≤ 1.0. Assessment of the reliability of measurements was performed by calculation of inter- and intraclass correlation coefficients. Inter- and intra-observer agreements were evaluated by measuring intraclass correlation coefficients (ICCs). The significance level for all tests was 5% (two-sided), and all data were analyzed using a commercially available software program (JMP version 15; SAS Institute Inc., Cary, NC, USA).

## Results

Characteristics of the volunteers are presented in Table 2.

**Table 2:** Characteristics of the study population.

Variables	Overall (n = 32)	Men (n = 16)	Women (n = 16)
	Mean ± SD (range)	Mean ± SD (range)	Mean ± SD (range)
Age, years	48.4 ± 11.5 (30–68)	48.4 ± 12.9 (30–68)	48.4 ± 10.2 (33–63)
BMI, kg/m^2^	22.5 ± 3.0 (16.7–30.6)	23.3 ± 3.4 (18.9–30.6)	21.7 ± 2.4 (16.7–25.7)
Height, cm	163.3 ± 7.7 (147.7–177.0)	168.1 ± 6.3 (155.2–177.0)	158.6 ± 6.0 (147.7–170.7)
Weight, kg	60.3 ± 10.8 (41.6–88.0)	65.9 ± 10.8 (47.6–88.0)	54.3 ± 7.5 (41.6–66.2)

*BMI* body mass index, *SD* standard deviation.

**Figure 3:**
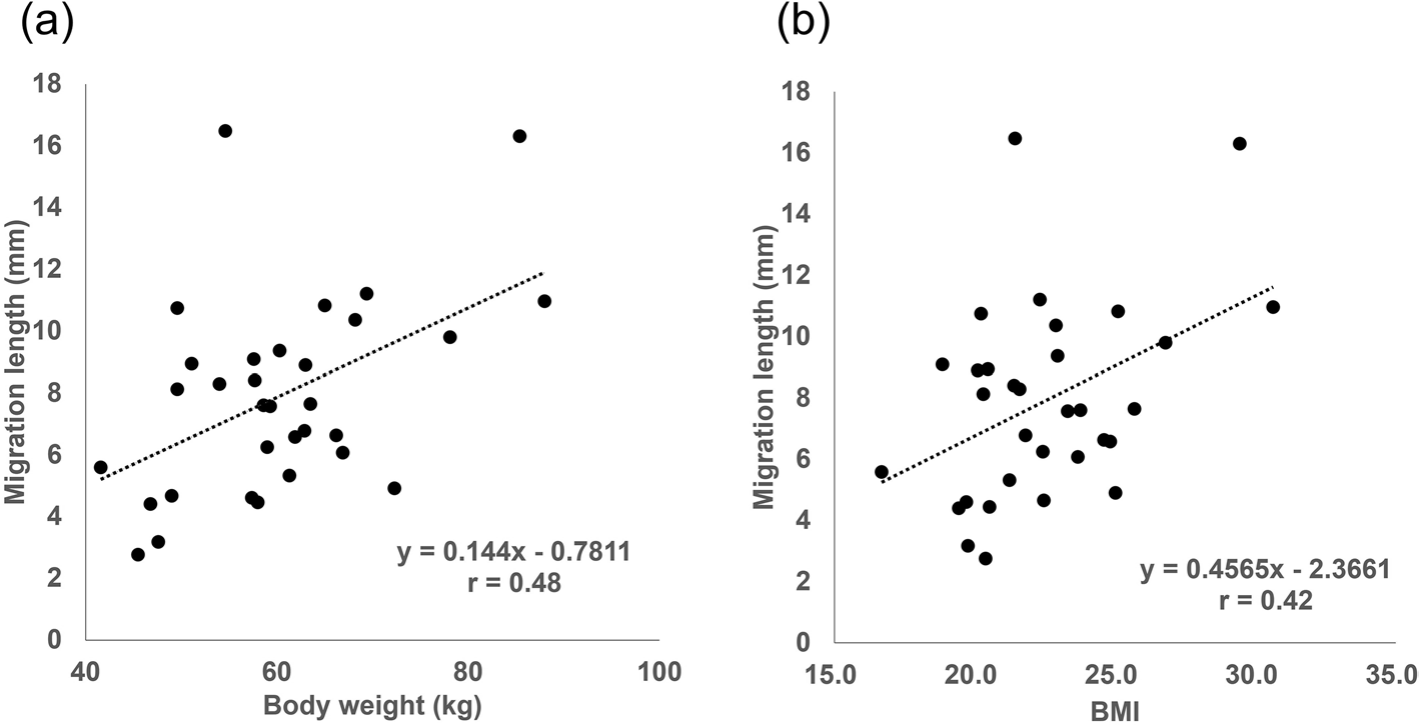
Scatter plots of correlation analysis. Scatter plots for the migration length of the tip of the coccyx versus body weight (**a**) and body mass index (BMI) (**b**), with linear regression lines (dotted lines). The migration length exhibited a moderate correlation with increased body weight (*r* = 0.48, *p* = 0.0054) and increased BMI (*r* = 0.42, *p* = 0.0163).

The mean straight lengths of the coccyx, sacrum, and sacrococcygeal segment; the mean coccygeal, sacral, and sacrococcygeal curvatures; and the mean differences between the standing and supine positions are shown in Table 3. The coccygeal straight length (mean difference 2.1 mm, [95% confidence interval {CI}, 1.2–2.9], *p* < 0.0001) and the sacrococcygeal straight length (mean difference 6.7 mm, [95% CI, 5.4–8.1], *p* < 0.0001) were significantly longer in the standing position than in the supine position. The sacrococcygeal angle (mean difference 10.0°, [95% CI, 7.9–12.1], *p* < 0.0001) and the intercoccygeal angle (mean difference 9.0°, [95% CI, 4.3–13.7], *p* = 0.0005) were significantly larger in the standing position than in the supine position. The lumbosacral angle (mean difference − 4.0°, [95% CI, − 4.9 to − 3.0], *p* < 0.0001) was significantly smaller in the standing position than in the supine position. There were no significant differences in the sacral straight length or the sacrococcygeal joint angle. The mean migration lengths of the tip of the coccyx were 0.15 mm (95% CI, − 0.82–1.13) to the left in the x-axis, 4.3 mm (95% CI, 3.12–5.53) backward in the y-axis, and 5.2 mm (95% CI, 4.15–6.29) downward in the z-axis in the standing position. The mean migration length of the tip of the coccyx was 7.9 mm (95% CI, 6.7–9.1), and all coccyges migrated backward and downward. The migration length exhibited a moderate correlation with increased body weight (*r* = 0.48, *p* = 0.0054) and increased BMI (*r* = 0.42, *p* = 0.0163) (Fig. 3). There was no significant difference in migration length between men and women (*p* = 0.486), and migration length was correlated with neither height (*r* = 0.31, *p* = 0.0873) nor age (*r* =  − 0.06, *p* = 0.756).

**Table 3:** Summary statistics for parameters in the standing and supine positions.

Parameters	Mean ± SD (range)		Difference between the standing and supine positions	Difference between men and women
	Standing	Supine	*p* value	*p* value
**Coccygeal straight length (mm)**				
All	37.8 ± 7.1 (20.0–50.7)	35.7 ± 7.0 (18.5–56.0)	*p* < 0.0001	
Men	38.9 ± 7.1 (25.9–48.7)	36.6 ± 6.1 (25.4–45.4)	*p* = 0.0014	*p* = 0.792
Women	36.7 ± 7.2 (20.0–50.7)	34.9 ± 7.9 (18.5–56.0)	*p* = 0.0126	
**Sacral straight length (mm)**				
All	111.0 ± 9.4 (89.4–135.4)	108.7 ± 16.5 (88.6–133.6)	*p* = 0.213	
Men	110.2 ± 8.8 (89.4–127.5)	106.0 ± 5.1 (88.6–126.7)	*p* = 0.0559	*p* = 0.068
Women	111.8 ± 10.1 (95.7–135.4)	111.4 ± 2.8 (92.8–133.6)	*p* = 0.734	
**Sacrococcygeal straight length (mm)**				
All	131.7 ± 11.2 (113.7–149.2)	125.0 ± 10.7 (107.8–143.3)	*p* < 0.0001	
Men	133.1 ± 2.8 (113.7–149.2)	126.3 ± 2.8 (109.4–143.3)	*p* < 0.0001	*p* = 0.865
Women	130.2 ± 2.8 (114.1–148.0)	123.7 ± 2.6 (107.8–142.3)	*p* < 0.0001	
**Lumbosacral angle (°)**				
All	21.1 ± 5.9 (5.7–30.3)	25.0 ± 4.9 (11.8–33.7)	*p* < 0.0001	
Men	20.7 ± 1.5 (8.0–30.3)	24.5 ± 4.5 (15.1–30.5)	*p* < 0.0001	*p* = 0.792
Women	21.5 ± 1.5 (5.7–30.0)	25.6 ± 5.6 (11.8–33.7)	*p* < 0.0001	
**Sacrococcygeal angle (°)**				
All	115.0 ± 10.6 (96.4–140.5)	105.0 ± 12.5 (78.5–138.4)	*p* < 0.0001	
Men	117.7 ± 10.6 (104.3–140.5)	107.1 ± 9.9 (96.0–126.2)	*p* < 0.0001	*p* = 0.792
Women	112.3 ± 10.2 (96.4–134.8)	102.8 ± 14.6 (78.5–138.4)	*p* < 0.0001	
**Sacrococcygeal joint angle (°)**				
All	159.6 ± 8.7 (144.5–178.2)	159.5 ± 10.7 (132.1–178.6)	*p* = 0.899	
Men	162.7 ± 7.3 (153.0–178.2)	163.2 ± 8.6 (143.0–178.6)	*p* = 0.734	*p* = 0.806
Women	156.6 ± 9.1 (144.5–174.0)	155.7 ± 11.6 (132.1–174.7)	*p* = 0.706	
**Intercoccygeal angle (°)**				
All	159.1 ± 15.2 (114.3–179.0)	150.1 ± 16.8 (116.1–176.9)	*p* = 0.0005	
Men	159.2 ± 14.8 (125.3–179.0)	151.1 ± 18.6 (116.1–176.9)	*p* = 0.0149	*p* = 0.749
Women	159.0 ± 16.0 (114.3–178.7)	149.1 ± 15.3 (121.6–175.2)	*p* = 0.0123	
**Migration length (mm)**				
All	7.9 ± 3.2 (2.8–16.5)			
Men	8.2 ± 3.3 (3.2–16.3)			*p* = 0.486
Women	7.6 ± 3.3 (2.8–16.5)

Differences in any coccygeal parameter between the standing and supine positions did not differ significantly between men and women. Analyses for parous and nulliparous women are described in the supplementary Table.

Inter- and intra-observer correlation coefficients were between 0.82 and 0.94 and between 0.91 and 0.97, respectively, for all quantitative measurements.

## Discussion

In this prospective study, we demonstrated that the coccyx became longer and straighter in the standing position, with the tip of the coccyx moving backward and downward in this position. Coccydynia has previously been reported to be associated with obesity^7^ and vaginal delivery^8^ and to be aggravated by standing and/or walking^6^. In our study, there were positive correlations between BMI, body weight, and the mobility of the coccyx. We believe our findings are important because, although we included only asymptomatic volunteers, the coccyx was shown to move in a similar manner to what can lead to coccydynia; therefore, our results may provide potentially important clues regarding the pathogenesis of coccydynia. Additionally, our study identified normal reference values for coccygeal measurements in the standing position using upright CT imaging.

Our study showed no significant differences in coccygeal parameters between men and women. In contrast, Karadimas et al. reported that coccydynia development is approximately four times more likely in women than in men^6^. In addition, other studies have demonstrated that the coccyx is significantly longer in male individuals while being more ventrally angulated in female individuals^10–12^. These findings may seem contradictory; however, this discrepancy may have resulted from the small number of volunteers in our study (only 16 male volunteers and 16 female volunteers). Therefore, these differences may not have been determined to statistically significant. Further research should be conducted to compare coccygeal parameters between men and women. Our study also showed that women with a history of vaginal deliveries tended to have a longer coccygeal migration length. However, the study design was too small to analyze the differences between parous and nulliparous women.

Some authors have investigated coccygeal movement using open dynamic MRI^3,7,25^ or plain radiography^2^. During dynamic MRI, images can be obtained when patients are either contracting or squeezing the pelvic muscles or when these muscles are relaxed. MRI can effectively evaluate pelvic floor morphology; however, dynamic MRI generally requires 15–30 min, whereas upright CT takes less than 3 min for two scout scans and one main scan^18^. In addition, dynamic MRI uses unnatural abdominal pressure during scanning, while upright CT imaging permits scanning in a natural position, as gravity is automatically applied in the vertical direction. In contrast, plain radiography can be performed more easily than CT imaging with less radiation exposure; however, evaluable parameters are restricted using this imaging modality.

This study had several limitations. First, our sample size was small and restricted to only a single Japanese center. Previous studies have suggested that racial differences do exist in the morphology and morphometry of the coccyx^9–16^. Thus, further studies including more ethic groups and larger patient populations are necessary. Second, our study focused solely on the standing and supine positions. However, since a previous study has shown that individuals with coccydynia show transient exacerbation of pain when standing up from a sitting position^7^, assessment in the sitting position is also important. Finally, although the observers independently evaluated images in a blinded and randomized manner, they may have recognized the positioning of subjects in some cases due to the presence or absence of a CT table. The inter- and intra-observer agreements (ICCs), however, were relatively high in this study.

## Conclusions

Our study demonstrated that the coccyx becomes longer and straighter in the standing position, with the tip of the coccyx moving backward and downward relative to the supine position. Furthermore, the migration length exhibited a moderate correlation with increased BMI and body weight.

## Supplementary Information

Supplementary Information

## Data Availability

The datasets generated during and/or analyzed during the current study are available from the corresponding author on reasonable request.
