# Supplementary material for: Three-dimensional evaluation of the coccyx movement between supine and standing positions using conventional and upright computed tomography imaging
Source: Sci Rep. 2021 Mar 25;11:6886. doi: 10.1038/s41598-021-86312-0 (PMC7994836; doi:10.1038/s41598-021-86312-0)
Supplement: Supplementary file 1 — Supplementary Information [file 41598_2021_86312_MOESM1_ESM.docx]

**Three-dimensional evaluation of the coccyx movement between supine and standing positions using conventional and upright computed tomography imaging**

Fumiko Yagi^1^, MD; Yoshitake Yamada^1^, MD, PhD; Minoru Yamada^1^, PhD; Yoichi Yokoyama^1^, MD; Kiyoko Mukai^1,2^, MD; Takehiro Nakahara^1^, MD, PhD; Keiichi Narita^1^, MD; Masahiro Jinzaki^1^, MD, PhD

^1^ Department of Radiology, Keio University School of Medicine, Tokyo, Japan

^2^ Department of Diagnostic Radiology, St. Luke's International Hospital, Tokyo, Japan

**Supplementary Table.** Computed tomography measurements of coccygeal parameters in parous and nulliparous women

| **Parameters** | **Mean ± SD (range)** | | | **Difference between the standing and supine positions** | **Difference between parous and nulliparous women** |
| --- | --- | --- | --- | --- | --- |
|  | **Standing** | | **Supine** | ***p*-value** | ***p*-value** |
| **Coccygeal straight length (mm)** |  | |  |  |  |
| All women (n = 16) | 36.7 ± 7.2 (20.0–50.7) | | 34.9 ± 7.9 (18.5–56.0) | *p* = 0.0126 |  |
| Parous women (n = 9) | 40.0 ± 5.2 (32.5–50.7) | | 38.1 ± 7.5 (30.4–56.0) | *p* = 0.1641 | *p* = 0.491 |
| Nulliparous women (n = 7) | 32.4 ± 7.4 (20.0–44.4) | | 30.7 ± 6.9 (18.5–42.0) | *p* = 0.0313 |  |
| **Sacral straight length (mm)** |  | |  |  |  |
| All women | 111.8 ± 10.1 (95.7–135.4) | | 111.4 ± 2.8 (92.8–133.6) | *p* = 0.734 |  |
| Parous women | 113.6 ± 10.3 (99.1–135.4) | | 112.2 ± 11.8 (92.8–133.6) | *p* = 0.484 | *p* = 0.169 |
| Nulliparous women | 109.4 ± 10.2 (95.7–126.7) | | 110.5 ± 11.3 (97.6–129.8) | *p* = 0.0156 |  |
| **Sacrococcygeal straight length (mm)** |  | |  |  |  |
| All women | 130.2 ± 2.8 (114.1–148.0) | | 123.7 ± 2.6 (107.8–142.3) | *p* < 0.0001 |  |
| Parous women | 133.3 ± 3.3 (124.3–146.9) | | 125.7 ± 3.4 (107.8–137.1) | *p* = 0.0078 | *p* = 0.244 |
| Nulliparous women | 126.3 ± 3.7 (114.1–148.0) | | 121.1 ± 3.9 (107.8–142.3) | *p* = 0.0156 |  |
| **Lumbosacral angle (°)** |  | |  |  |  |
| All women | 21.5 ± 1.5 (5.7–30.0) | | 25.6 ± 5.6 (11.8–33.7) | *p* < 0.0001 |  |
| Parous women | 22.1 ± 2.3 (12.8–27.0) | | 25.8 ± 1.8 (19.8–31.4) | *p* = 0.0039 | *p* = 0.597 |
| Nulliparous women | 20.6 ± 2.6 (5.7–30.0) | | 25.3 ± 2.1 (11.8–33.7) | *p* = 0.0156 |  |
| **Sacrococcygeal angle (°)** |  | |  |  |  |
| All women | 112.3 ± 10.2 (96.4–134.8) | | 102.8 ± 14.6 (78.5–138.4) | *p* < 0.0001 |  |
| Parous women | 110.4 ± 8.7 (96.4–126.0) | | 99.7 ± 4.5 (78.5–124.1) | *p* = 0.0039 | *p* = 0.672 |
| Nulliparous women | 114.8 ± 12.2 (101.0–134.8) | | 106.8 ± 5.5 (93.2–138.4) | *p* = 0.0313 |  |
| **Sacrococcygeal joint angle (°)** |  | |  |  |  |
| All women | 156.6 ± 9.1(144.5–174.0) | | 155.7 ± 11.6 (132.1–174.7) | *p* = 0.706 |  |
| Parous women | 156.6 ± 9.3 (146.7–174.0) | | 153.5 ± 12.4 (132.1–168.8) | *p* = 0.426 | *p* = 0.244 |
| Nulliparous women | 156.5 ± 9.6 (144.5–169.6) | | 158.7 ± 10.5 (145.3–174.7) | *p* = 0.813 |  |
| **Intercoccygeal angle (°)** |  | |  |  |  |
| All women | 159.0 ± 16.0 (114.3–178.7) | | 149.1 ± 15.3 (121.6–175.2) | *p* = 0.0123 |  |
| Parous women | 153.6 ± 5.1 (114.3–175.2) | | 147.3 ± 14.7 (130.8–171.8) | *p* = 0.195 | *p* = 0.368 |
| Nulliparous women | 166.0 ± 5.8 (156.5–178.7) | | 151.5 ± 16.9 (121.6–175.2) | *p* = 0.0313 |  |
| **Migration length (mm)** |  |  | |  |  |
| All women | 7.6 ±3.3 (2.8–16.5) | | |  |  |
| Parous women | 8.7 ± 3.6 (4.4–16.5) | | |  | *p* = 0.169 |
| Nulliparous women | 6.1 ± 2.1 (2.8–8.1) | | |  |  |

SD, standard deviation
